# An intervention to promote physical activity and self-management in people with stable chronic heart failure The Home-Heart-Walk study: study protocol for a randomized controlled trial

**DOI:** 10.1186/1745-6215-12-63

**Published:** 2011-03-02

**Authors:** Hui Y Du, Phillip J Newton, Robert Zecchin, Robert Denniss, Yenna Salamonson, Bronwyn Everett, David C Currow, Peter S Macdonald, Patricia M Davidson

**Affiliations:** 1 Curtin Health Innovation Research Institute, Curtin University, Sydney, (39 Regent Street), Chippendale, (2008), Australia; 2 Centre for Cardiovascular & Chronic Care, Faulty of Nursing, Midwifery & Health, University of Technology Sydney, (15 Broadway), Ultimo, (2007), Australia; 3 Cardiac Assessment and Education Program, Sydney West Area Health Service, (Darcy Rd), Westmead, (2145), Australia; 4 Westmead Specialist Medical Ctr, Westmead and Blacktown Hospitals & University of Western Sydney, (202/151-155 Hawkesbury Rd), Westmead, (2145), Australia; 5 School of Nursing & Midwifery,University of Western Sydney, (Cr Narellan Rd & Gilchrist Dr), Campbelltown, (2560), Australia; 6 Faculty of Nursing, Midwifery & Health, University of Technology Sydney, (15 Broadway), Ultimo, (2007), Australia; 7 Discipline of Palliative and Support Service, Flinders University, (Sturt Rd), Bedford Park, (5001), Australia; 8 St Vincents Hospital, Darlinghurst. Victor Chang Cardiac Research Institute, (405 Liverpool St), Darlinghurst, (2010), Australia; 9 St Vincents & Mater Health, Centre for Cardiovascular & Chronic Care, Faculty of Nursing, Midwifery & Health, University of Technology Sydney, (15 Broadway), Ultimo, (2007), Australia

## Abstract

**Background:**

Chronic heart failure (CHF) is a chronic debilitating condition with economic consequences, mostly because of frequent hospitalisations. Physical activity and adequate self-management capacity are important risk reduction strategies in the management of CHF. The Home-Heart-Walk is a self-monitoring intervention. This model of intervention has adapted the 6-minute walk test as a home-based activity that is self-administered and can be used for monitoring physical functional capacity in people with CHF. The aim of the Home-Heart-Walk program is to promote adherence to physical activity recommendations and improving self-management in people with CHF.

**Methods/Design:**

A randomised controlled trial is being conducted in English speaking people with CHF in four hospitals in Sydney, Australia. Individuals diagnosed with CHF, in New York Heart Association Functional Class II or III, with a previous admission to hospital for CHF are eligible to participate. Based on a previous CHF study and a loss to follow-up of 10%, 166 participants are required to be able to detect a 12-point difference in the study primary endpoint (SF-36 physical function domain).

All enrolled participant receive an information session with a cardiovascular nurse. This information session covers key self-management components of CHF: daily weight; diet (salt reduction); medication adherence; and physical activity. Participants are randomised to either intervention or control group through the study randomisation centre after baseline questionnaires and assessment are completed. For people in the intervention group, the research nurse also explains the weekly Home-Heart-Walk protocol. All participants receive monthly phone calls from a research coordinator for six months, and outcome measures are conducted at one, three and six months. The primary outcome of the trial is the physical functioning domain of quality of life, measured by the physical functioning subscale of the Medical Outcome Study Short Form -36. Secondary outcomes include physical functional capacity measured by the standard six minute walk test, self-management capacity, health related quality of life measured by Medical Outcome Study Short Form -36 and Minnesota Living With Heart Failure Questionnaire, self-efficacy and self-care behaviour.

**Discussion:**

A self-monitoring intervention that can improve individual's exercise self-efficacy, self-management capacity could have potential significance in improving the management of people with chronic heart failure in community settings.

**Trial Registration:**

Australian New Zealand Clinical Trial Registry 12609000437268

## Background

Chronic heart failure (CHF) is a prevalent and debilitating condition and is most common in the elderly population. Apart from the symptoms that individuals suffer and the burden on their families, this condition poses a substantial economic burden for the wider society[1,2]. It is estimated that CHF costs Australia AUD$1 billion annually, with the bulk of these costs related to hospitalization[3]. Studies have shown that at least 40% of readmissions to hospital for CHF are due to non-compliance with treatment recommendations and inadequate patient information[4]. Although physical activity is important and beneficial in the management of CHF, many individuals consider that adhering to physical activity recommendations is harder compared to implementing strategies related to medications, dietary modifications or fluid restriction[5].

The theoretical concept underpinning adherence to physical activity is self-efficacy, which is the central concept of self-management[6]. Individuals' exercise self-efficacy determines, in part, an individual's ability to initiate and sustain an exercise program[6]. Because the benefits of physical activity are often not immediately felt, this can be a barrier to sustainable behavior change. Without prompted monitoring of progress, the importance and usefulness of physical activity is likely to be overlooked. In addition, while many people with CHF are managing in the community, monitoring of physical functional capacity is most often undertaken in a clinical environment where it requires well-trained staff and equipment. Hospitalisation for CHF can occur when individuals fail to recognise the signs and symptoms of deteriorating physical function. Without adequate knowledge and support, signs and symptoms such as shortness of breath, weight gain or increasing fatigue can be disregarded[7]. Self-monitoring is one important but often under-utilised self-management behaviors[8,9].

With the aim of assisting individuals and clinicians to monitor and promote physical activity, we developed the Home-Heart-Walk (HHW). The HHW has been developed based on the principle of self-efficacy and the mechanistic elements of the 6MWT[10]. The HHW is a self-administered, modified 6MWT. We chose to base the HHW protocol on the 6MWT over other exercise tests because of its simple, safe and inexpensive characteristics and the ability to consider data sharing in the future. The HHW program is a structured, self-monitoring exercise program, which incorporates regular HHW and telephone follow-up. After developing the HHW prototype, we have assessed the correlation of the HHW distance and the 6MWT distance in a group of 13 healthy adult (age 23 to 59) and the reliability and feasibility in a group of 29 cardiac rehabilitation participants[11]. The correlation between the two tests distances was r = 0.81[11]. The intra-class correlation coefficient of the test distance over a 7 day period was 0.98 and the inter-rater correlation was almost perfect (r = 0.99)[12]. Given the impact that regular monitoring of the HHW could have on the management of CHF, a randomized controlled trial to assess the ability of the HHW to promote self-management and exercise self-efficacy was devised.

### Intervention

**Figure 1:**
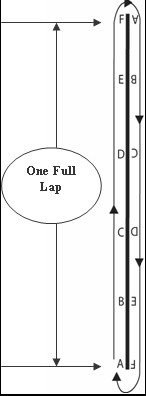
**Illustration of the walking track**. Source:(Du, H. et al. 2009).

Based on a 6MWT literature review, [10] our research team has modified the 6MWT to a home-based, self-administered monitoring tool for functional capacity. According to American Thoracic Society 6MWT guideline, a 30 meter long walking track is required. However, a 30 meter long walking track is rarely feasible in a home environment. In the HHW, walking is performed alongside a 5-meter length of rope (marked in 1-meter gradations) on a flat hard surface free of obstacles. The purpose of the HHW is to cover as much distance as possible in 6 minutes (Figure 1). In parallel with the 6MWT, stopping and resting were allowed during the HHW test. However, standard encouragement using the American Thoracic Society guideline is not used during the HHW protocol. As a self-administered tool, a lap counter was used to assist the individual to count the number of full laps walked during the test. A timer with a countdown function was used to time the six minutes. Investigators calculate the distance covered during the HHW test after the study is completed (Laps walked × distance (in meters) per lap **+ **partial lap).

The HHW program is based on a ***SMART*** concept which is Simple, Measurable, Achievable, Realistic/relevant and Timed, that assists to improve individuals exercise self-efficacy[11]. When telephone support is incorporated, the Home-Heart-Walk program could be an effective intervention to assist people to recognize and response to any functional changes, thereby improving their self-management capacity.

### Aim

The study aims to evaluate the effect of the HHW program on exercise self-efficacy and self-management in people with stable CHF. The rationale for this approach is ***Firstly***, this approach seeks to promote physical activity-any bodily movement produced by skeletal muscles that result in energy expenditure[13] using a method that is accurate and reproducible. ***Secondly***, it provides a measurement tool whereby individuals and their clinicians can monitor their condition. ***Thirdly*, **it may increase self-efficacy for physical activity which can likely be translated to other self-care behaviours [14,15] and ***fourthly***, improve health related quality of life (HRQoL) which includes the overall impact of a medical condition on the physical, mental, and social well-being of an individual[16].

## Study design and duration

The HHW study is a multicentre, non-blinded, parallel arm, randomized controlled trial. For each individual, the study will last for 6 months, and recruitment to the whole study is expected to be completed within twelve months.

### Selection criteria

The diagnosis of CHF is based on the Australian National Heart Foundation/Cardiac Society of Australia and New Zealand guidelines[17]. Individuals with diagnosed CHF; with a previous hospitalisation for CHF; in New York Heart Association Functional Class (NYHA-FC) II or III and approved by their clinicians for physical activity will be invited to participate. Individuals with unstable angina pectoris; recent unexplained syncope; resting heart rate greater than 120 beats per minute; inability to perform the 6MWT; failure to give informed consent or significant cognitive impairment will be excluded from this study.(Table 1)

**Table 1:** Inclusion and exclusion criteria

Inclusion Criteria	Exclusion Criteria
Have had a previous hospitalisation for heart failure	Unstable angina pectoris
In New York Heart Association-Functional Class II or III	Recent unexplained syncope
Approval from responsible clinician to participate in the Home-Heart-Walk program	Failure to give informed consent
	Resting HR greater then 120
	Inability to perform 6MWT
	Significant cognitive impairment

### Recruitment and randomization

#### Sample Size Calculation

Based on a previous CHF study, to detect a 12-point difference in physical function on the Short Form-36 with a standard deviation of 20 points, we estimated a sample size of 74 per group is needed with a 2-sided 5% significance level and 95% power. When taking into account a loss to follow-up of 10%, 166 participants (83 per group) will be required.

#### Recruitment

Potential participants are identified at cardiac wards and cardiac clinics at each of the study sites. Research nurses explain the HHW study in detail to individuals who are interested in the study and agree to be contacted by the research nurse. Once consented, participants will undergo a screening phase, which includes review of their medical records, resting blood pressure and heart rate for assessment of eligibility. The research team keeps a study log where all screened potential participants are recorded and the reason if they are not enrolled in the study. These data will provide a breakdown of the overall pool of patients we have accessed and the ability to identify reason for non-participation. The study design allows for a period of 6 months follow-up per participant.

#### Consent Form

Participants are asked to provide written informed consent to participate in this study after reading the Information Sheet and having any questions answered appropriately.

#### Randomization

Participants are randomly assigned to either the intervention or the control group after completion of baseline questionnaire and assessment. Randomization is undertaken using a central phone randomization centre using computer generated random numbers.

#### Blinding

As the HHW is a self-administered intervention, blinding of the participant and the researcher who conduct telephone follow-up is not possible. However, three and six month follow-ups and data analysis is undertaken by an assessor blinded to treatment allocation.

## Study measurements

Demographic information, clinical status, functional and general health status as well as their self-management capacity as shown in Table 2 will be collected for participants in both the intervention and control group. A brief physical examination is carried out on all participants to collect the following data: height; weight; waist circumference; hip circumference; heart rate; blood pressure; pulse oximetry; and a cardio-respiratory examination.

**Table 2:** Study measurements for both intervention and control group:

**Demographic profile**:	Age, sex, marital status, social support, education, ethnicity, health care utilization
**Clinical status:**	Type, presumed cause and duration of CHF, NYHA Functional Class, current pharmacological and non-pharmacological treatment, waist and hip circumference, and existing blood test results, existing echocardiography results

**Functional/general health status:**	The Six Minute Walk Test distance, National Physical Activity Prescription-Likert scale and SF-36, Minnesota Living With Heart failure Questionnaire

**Self-management:**	Bandura's Exercise Self-efficacy Scale; The European Heart Failure Self-care Behaviour Scale

### Study instruments

*The 6MWT*: distance walked on a standard 6MWT are used to measuring physical functional capacity; [18]*European Heart Failure Self-care Behaviour Scale *is used to measure self-care capacity;[19] The *Minnesota Living With Heart Failure Questionnaire *and Medical Outcome Study Short Form-36 are used to assess HRQoL; [20] The individual's level of physical activity is measured by using the National Physical Activity Prescription Scale; [13] and Bandura's Exercise Self-efficacy Scale is used for measuring level of exercise self-efficacy[21].

## procedure

**Figure 2:**
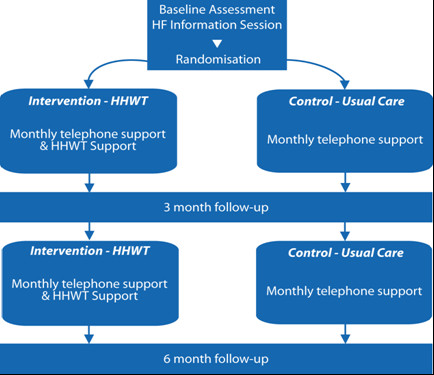
**Home-Heart-Walk study schemata**.

Individuals who participate in the study undergo a baseline physical assessment, a standard 6MWT, and receive an information session from a cardiac nurse on HF self-management. They are also provided with National Heart Foundation Consumer Resource Guide[22]. A summary of study schemata is presented as Figure 2.

### Intervention group

The intervention group receives instructions on undertaking the HHW by the research nurse. They are given specific instructions on the HHW and the research nurse observes their competency in the technique. Participants are asked to perform the HHW daily for the following week to gain proficiency. On Day 2 the research nurse calls them to clarify their understanding of the procedure. The research nurse calls participants on Day 7 to clarify any concerns and answers any questions. Subsequently, participants in the intervention group are asked to undertake the HHW at least weekly, and record this information in the HHW program diary. A monthly phone call is organized with the participant.

### Control group

Apart from an identical baseline information session, the control group will also receive a monthly telephone call from the research nurse.

#### Telephone follow-up

Participants in both intervention and control group will receive monthly telephone calls from the research nurse. During the telephone call, the research nurse will obtain participants HHW distance for the past month for the intervention group. Control group will just have a general phone call to check if there is any change in their condition, or any hospitalisation during the past month.

The study procedure for both intervention and control group is summarized in Table 3

**Table 3:** Study procedures for both intervention and control group.

Measure	Baseline	3 Months	6 months
Clinical history, physical assessment and health care utilisation	X	X	X
Sociodemographic data	X		
Self-Efficacy [21]	X	X	X
6MWT[18]	X	X	X
SF-36[23]	X	X	X
EHFScBS[19]	X	X	X
MLWHFQ[20]	X	X	X
Physical activity scale[13]	X	X	X

## Study endpoints

### Primary endpoint

#### Health outcome measure

The SF-36 will be used to assess the HRQoL[23]. Although the primary outcome will be physical functioning subscale of the SF-36, all eight subscales of the SF-36 will be measured. Items scores related to each subscale are coded, summed and scaled from 0 (worst possible health state) to 100 (best possible health state).

#### Secondary endpoints

Secondary endpoints include: distance walked on a standard 6MWT[18] European Heart Failure Self-care Behaviour Scale;[19] Minnesota Living With Heart Failure Questionnaire; [20] National Physical Activity Prescription Scale; [13] and Bandura's Exercise Self-efficacy Scale [21]. These measurements are administered at baseline, three and six month follow-up.

### Adverse event monitoring

For the purpose of this study a ***Serious Adverse Event*** will be defined as any untoward medical occurrence resulting in hospitalisation or prolongation of hospitalisation, or is life-threatening, or results in death or disability[24]. ***Adverse events*** will be defined as any untoward occurrences in study participants, potentially related to implementation of the study protocol. All serious adverse events will be reported to the Coordinating Centre and the Human Research Ethics Committee within 24 hours and adverse events within 7 days as required.

### Data analysis

Survey data will be coded and analysed using the Statistical Package for the Social Sciences computer statistical software. Continuous data with normal distribution will be analysed using independent t test and Mann-Whitney U test for non-normally distributed data. Association between categorical data will be analysed using Chi-square test. When comparing change of SF-36 subscale scores over time within groups; between baseline and 6-month follow-up, the Wilcoxon signed ranks test will be used. A *p *value of < 0.05 will be considered statistically significant and all tests will be two tailed. Data analysis will be supervised by a statistician who is not involved in screening, recruitment and follow-up of study participants.

### Ethical issues

The study will be conducted according to the principle of the declaration of Helsinki (version 2004) and the National Health and Medical Research Council Guidelines for the ethical conduct of clinical research and ethical approval has been obtained from the Curtin University [approval number HR 170/2008] and the relevant clinical sites [St Vincent's Hospital, approval number 08/SVH/77].

## Discussion

This HHW study is a novel approach to self-management in CHF. Low adherence to health recommendations suggest the need to explore ways of better promoting adherence to clinical advice and develop innovative interventions for monitoring and managing people with CHF in community settings. If evaluated as being successful, the HHW protocol can be readily incorporated into the community based management of CHF. In particular it has the potential to reach the subgroup of CHF population who have limited physical functional capacity and are not appropriate for higher intensity physical activity. At the same time, this self-monitoring intervention if shown to be beneficial would also assist in managing people living in areas where clinical based, supervised programs are not easily accessible.

The use of self-reported instruments could be a potential limitation of this study, because the risk of over reporting or under reporting from the participants and is likely to be evenly distributed between the randomized groups. However, this is inevitable with any kind of study using self-reporting instruments. The study instruments and scoring will be discussed when reporting study findings. Enrolment of the 166 participants will continue through 2010. After recruitment of the 166 participants, the baseline characteristics of the participants will be analysed. Outcome assessment is projected to be completed by mid 2011.

## Abbreviations

CHF: Chronic heart failure; SF-36: Medical Outcome Study Short Form-36; MLWHF: Minnesota Living With Heart Failure Questionnaire; HHW: Home-Heart-Walk; 6MWT: 6-minute walk test; HRQoL: Health related quality of life; NYHA-FC: New York Heart Association-Functional Class; EHFScBS: European Heart Failure Self-care Behaviour Scale

## Competing interests

The authors declare that they have no competing interests.

## Authors' contributions

PMD conceived the study, HD, PJN, RZ, BE, ARD, YS, DC, PM, PMD participated in the design of the study, writing of the study protocol and the manuscript. All authors read and approved the final manuscript.
